# Sleep patterns, physical activity and glycemic control in newly diagnosed type 2 diabetes patients from a joint perspective: a cross-sectional study

**DOI:** 10.3389/fendo.2026.1845188

**Published:** 2026-06-17

**Authors:** Yuan Xu, Ruiying Jin, Jiaheng Pang, Feng Chen, Siyu Dai

**Affiliations:** 1 School of Clinical Medicine and The Affiliated Hospital, Hangzhou Normal University, Hangzhou, China; 2 The Li Ka Shing (LKS) Faculty of Medicine, The University of Hong Kong, Hong Kong, Hong Kong SAR, China; 3 Faculty of Medicine, The Chinese University of Hong Kong, Hong Kong, Hong Kong SAR, China

**Keywords:** glycemic control, interaction effects, newly diagnosed T2DM, physical activity, sleep duration

## Abstract

**Background:**

The newly diagnosed stage of type 2 diabetes mellitus (T2DM) represents a “critical window” for delaying disease progression through lifestyle intervention. Insufficient physical activity and sleep disorders are both known risk factors for metabolic disorders. However, evidence regarding the independent and combined effects of sleep patterns and physical activity on weekdays and weekends in newly diagnosed patients is currently lacking.

**Methods:**

This study employed a cross-sectional design and included 340 patients with newly diagnosed type 2 diabetes who had not received any hypoglycemic medical treatment previously. The average sleep duration on weekdays and weekends was collected through standardized sleep diaries, and the physical activity level was calculated in metabolic equivalents (METs) using the long-form International Physical Activity Questionnaire (IPAQ). Multivariate linear regression models and generalized additive models were performed to analyze the associations between sleep, physical activity, fasting blood glucose, and glycated hemoglobin. A combined grouping variable of sleep and physical activity was constructed to evaluate the potential interaction effects.

**Results:**

We found that there was a statistically significant U-shaped association between sleep duration on weekdays and fasting blood glucose (p < 0.05), with the optimal point at around 6.92 hours. Physical activity could be a protective factor for glycemic control. Compared with the low-activity group, both moderate-intensity and high-intensity activities were significantly associated with lower fasting blood glucose (β = -2.76 and -3.22) and glycated hemoglobin (β = -1.50 and -2.01) levels, but there was no significant difference observed when comparing the moderate- and high-intensity activity groups. The interaction analysis of the groups shown that moderate to high-intensity physical activity may have promising glycemic control effects. On weekdays, “moderate sleep & high physical activity” (β = -3.57, p < 0.001) and on weekends, “long sleep & high physical activity” (β = -4.25, p < 0.001) respectively showed a potential notable glycemic control benefits.

**Conclusion:**

In newly diagnosed type 2 diabetes patients, maintaining a sleep duration of approximately 7 hours​ on workdays and engaging in at least moderate-intensity physical activity are associated with​ better early glycemic control. Moderate- to high-intensity physical activity is crucial and may partially offset the adverse glycemic effects of insufficient sleep.

## 1 Introduction

Diabetes mellitus (DM) is a metabolic disorder characterized by chronically elevated blood glucose levels due to impaired insulin secretion and insulin action, and can have various causes. Type 2 diabetes mellitus (T2DM), characterized by insulin resistance and relative insulin deficiency (1), is the most common form. Cases of T2DM account for approximately 96.0% of all diabetes cases worldwide (2). In 2023, the number of people with diabetes in China stood at approximately 233 million, representing an increase of nearly 50% in age-standardized prevalence compared to 2005 (3). According to the latest data from the International Diabetes Federation (IDF), the total number of adults with diabetes worldwide is projected to reach 853 million by 2050 (4). Studies show that diabetes reduces life expectancy by 4 to 10 years among people aged 40 to 60 and increases the risk of death from cardiovascular disease, kidney disease, or cancer by a factor of 1.3 to 3.0. At the same time, diabetes significantly increases the risk of non-traumatic lower limb amputation and blindness (5, 6). The “Chinese Guidelines for the Prevention and Treatment of Diabetes (2024)”, the “five key components” of intervention recommended by the International Diabetes Federation (IDF), and the latest clinical recommendations from the American College of Lifestyle Medicine (ACLM) all emphasize that lifestyle interventions are cornerstones of diabetes management​ (7–9). Thus, implementing early lifestyle interventions is essential for glycemic management in patients with diabetes.

The term “newly diagnosed patients with T2DM” refers to individuals who have been definitively diagnosed with T2DM within the past year and who have not yet undergone systematic glucose-lowering therapy (10). Studies have shown that initiating short-term intensive insulin therapy in the early stages of T2DM can promote glycemic remission (11). Similarly, lifestyle-focused interventions could help mitigate or even “reverse” carbohydrate metabolism disorders (12, 13). A study of patients with newly diagnosed T2DM showed that they adhered better to lifestyle changes than to medication (14). Previous studies have shown that effective glycemic control during the first year following diagnosis can significantly reduce the risk of long-term complications and death in patients (15, 16). It is therefore essential to implement active and intensive glycemic control in patients with T2DM from the time of diagnosis.

According to current guidelines, a glycated hemoglobin (HbA1c) level of ≥ 6.5% is one of the diagnostic criteria for diabetes. Using this criterion, in conjunction with fasting plasma glucose (FPG ≥ 7.0 mmol/L), improves diagnostic accuracy (7). Among the various factors influencing glycemic control, numerous observational studies shown that regular physical activity (PA) helps reduce the risk of diabetes-related complications (17–19). However, rare studies have examined the specific level of PA required for effective glycemic control in patients with T2DM. Similarly, it has been demonstrated that sleep, as a modifiable lifestyle factor, is significantly associated with metabolic function in patients with T2DM (20). However, the influence of sleep schedules on weekdays and weekends on this association has not yet been clearly established. Furthermore, data specific to patients newly diagnosed with T2DM remains insufficient.

The current study therefore focuses on patients newly diagnosed with T2DM and examines the independent and interactive effects of two modifiable lifestyle factors—sleep habits and physical activity—on glycemic control, with the goal of providing a foundation for the development of early management protocols tailored to this population.

## 2 Methods

### 2.1 Data sources and inclusion criteria

This cross-sectional study was conducted between January 2024 and June 2024 at The Affiliated Hospital of Hangzhou Normal University in China. The study protocol was approved by the University committee (Approval 2024 (e2)-ks-075), and all participants provided written informed consent. Participants were eligible if they were adults (aged ≥18 years) with newly diagnosed T2DM, whose diagnosis was established in strict accordance with the relevant criteria of the “National Guide for the Prevention and Management of Diabetes in Primary Care (2022)”: fasting plasma glucose (FPG) ≥ 7.0 mmol/L, or 2-hour glucose level during an oral glucose tolerance test (OGTT) ≥ 11.1 mmol/L, or glycated hemoglobin (HbA1C) ≥ 6.5%. These criteria were accompanied by symptoms typical of diabetes (such as excessive thirst, increased appetite, polyuria, and unexplained weight loss) (21). The exclusion criteria were as follows: (1) previously been diagnosed with T2DM; (2) with history of psychiatric or cognitive disorders; (3) severe organic disease or physical disability; (4) pregnancy or breastfeeding; (5) lack of HbA1c data; and (6) suspected type 1 diabetes. A total of 340 patients with T2DM were ultimately included in the study.

### 2.2 Collection of sleep duration data

We collected all data on sleep duration using a standardized, validated questionnaire. The questionnaire on sleep duration was referred to the international Sleep Diary which has been adopted widely and shown to with good reliability and validity (22). The questionnaire was distributed and then fulfilled by the patient under the supervision of trained medical and research staff. Participants were asked to record their bedtimes and wake-up times from Monday to Friday as well as on Saturday and Sunday. We then calculated the average sleep duration on weekdays and weekends accordingly. Responses that were unclear, imprecise, or contradictory were verified, corrected, and completed on the spot to avoid any memory bias or errors in completion. Once the questionnaires have been collected, they are immediately checked, and invalid questionnaires are discarded to ensure the authenticity, accuracy, and reliability of the data. Finally, based on the American Diabetes Association’s recommendations regarding sleep duration, valid participants were divided into three groups: short sleep (≤6 h/day), moderate sleep (6–8 h/day), and long sleep (>8 h/day) (23).

### 2.3 Collection of PA-related metrics

We collected all data related to physical activity by combining a standardized physical activity questionnaire with participants’ self-reports. All questionnaires were distributed by medical and nursing staff who had received specialized training and completed under their supervision. We adopted the long-form International Physical Activity Questionnaire (IPAQ) (24). This questionnaire measured an individual’s physical activity levels in four aspects: work, transportation, household chores, and leisure. It included information on the frequency, duration, and intensity of the activities (such as walking, moderate intensity, and high intensity) and there were 27 items in total. According to the IPAQ scoring protocol, the activity levels could be converted into metabolic equivalents (METs-min/week), and the MET calculation was based on the standards of IPAQ (24). For each aspect (such as transportation, housework, etc.), we calculated the MET-min/week values of the included walking, moderate-intensity and vigorous-intensity activities respectively. The formula was: MET-min/week of a certain type of activity = corresponding MET coefficient × minutes per day × days per week. Finally, we add up the total scores of all fields. During data collection, medical staff explained in detail to participants the meaning of each questionnaire section and guided them to accurately report their physical activities information based on their actual habits, thereby ensuring the accuracy and completeness of the data. In line with the World Health Organization (WHO) recommendations on physical activity for adults (25), participants were divided into three groups as follows: low level of physical activity (< 600 MET-min/week), moderate level of physical activity (600–1,200 MET-min/week), and high level of physical activity (≥ 1,200 MET-min/week).

### 2.4 Collection of covariates

The covariates considered fall into three main categories: sociodemographic characteristics (gender, age, educational level, occupation etc.), lifestyle (smoking, alcohol consumption etc.), as well as physical examination findings and biochemical parameters (BMI, systolic blood pressure, diastolic blood pressure, total cholesterol, triglycerides, LDL). Sociodemographic and lifestyle data were collected using a standardized structured questionnaire. All procedures are carried out by medical staff and researchers who have undergone specialized training, in strict accordance with standardized protocols, to ensure the authenticity, accuracy, and completeness of the covariate data.

Biochemical-related indicators were objectively measured through blood sampling. Data from medical examinations (systolic blood pressure, diastolic blood pressure, BMI (BMI = weight in kg/height in m²) were measured and collected by specialized researchers who had undergone standardized training, in strict accordance with established procedures. Prior to the collection of biochemical parameters (total cholesterol, triglycerides, LDL cholesterol): all participants were instructed to fast for at least 8 hours the night before. The following morning, a blood sample was drawn from the antecubital vein in a standardized setting. The samples were immediately transported to the clinical laboratory of the affiliated hospital, where specialized, trained researchers performed analyses using standardized procedures and clinical equipment. The analysis was conducted strictly in accordance with the clinical laboratories’ standard operating procedures to ensure the accuracy and reliability of the results.

### 2.5 Statistical analysis

Normally distributed data were reported as the mean ± standard deviation; non-normally distributed data were described using the median (interquartile range), while categorical variables were presented as frequencies (percentages). Differences between groups were assessed using the Kruskal-Wallis test, followed by Dunn-Bonferroni *post-hoc* tests. Multivariate linear regression analysis was performed to explore the associations between sleep, PA, and glycemic control. A Generalized Additive Model (GAM) was used to conduct supplementary analysis of the nonlinear relationship between sleep and blood glucose. To assess the interaction effect between sleep and physical activity, we combined the two into nine categorical variables (3 sleep and 3 activity levels). Using “short sleep & low PA” as the reference group, we included these combinations in a multiple linear regression model to analyze differences in blood glucose levels between the other combinations and the reference group. We constructed progressively adjusted regression models: Model 1 was unadjusted; Model 2 was adjusted for gender, age, education level, occupation, smoking history, and alcohol consumption history; Model 3 further included physical examination and biochemical indicators as confounders, including BMI, systolic blood pressure, diastolic blood pressure, total cholesterol, triglycerides, and LDL-cholesterol. Finally, two sensitivity analyses were conducted to test the robustness of the results by removing extreme sleep data and adopting new MET classification criteria. All regression analyses reported unstandardized regression coefficients (β), 95% confidence intervals (95% CI), and P-values to clarify the strength of association and statistical significance between each independent variable and the dependent variable. To ensure the robustness of the statistical models, the following diagnostics were performed. For the multiple linear regression models, multicollinearity was assessed using the variance inflation factor (VIF), with a VIF < 5 considered indicative of no substantial multicollinearity. For the GAM, the gam check function was used to evaluate the adequacy of the smooth terms and residual patterns. All data were organized and analyzed using SPSS 27.0.1 and R (version 4.10). All statistical tests were conducted at a significance level of α = 0.05, and P < 0.05 was considered statistically significant.

## 3 Results

### 3.1 Demographic characteristics

The baseline characteristics of the study population are presented in **Table 1**. A total of 340 participants were finally included (215 non-manual workers and 125 manual workers). There were significant differences between the two groups in terms of age, education level, smoking, drinking, and physical activity level (p < 0.05). No significant differences were observed between the two groups in terms of gender, past medical history, and physical indicators (p > 0.05).

**Table 1:** Baseline characteristics of participants by occupational type.

Characteristic category	Variables	Total (N=340)	Non-manual workers (N=215)	Manual workers (N=125)	P value
Demographic characteristics	Gender, n (%)				0.566
	Female	96 (28.2)	63 (29.3)	33 (26.4)	
	Male	244 (71.8)	152 (70.7)	92 (73.6)	
	Age grouping, n (%)				0.006
	<30 years	38 (11.2)	32 (14.9)	6 (4.8)	
	30–45 years	142 (41.8)	80 (37.2)	62 (49.6)	
	>45 years	160 (47.1)	103 (47.9)	57 (45.6)	
	Education, n (%)				<0.001
	Illiterate	5 (1.5)	3 (1.4)	2 (1.6)	
	Primary school	49 (14.4)	29 (13.5)	20 (16.0)	
	Junior high school	84 (24.7)	34 (15.8)	50 (40.0)	
	Senior high school	74 (21.8)	40 (18.6)	34 (27.2)	
	Junior college	58 (17.1)	47 (21.9)	11 (8.8)	
	Bachelor or above	70 (20.6)	62 (28.8)	8 (6.4)	
Lifestyle	Smoking, n (%)				<0.001
	No	240 (70.6)	167 (77.7)	73 (58.4)	
	Yes	100 (29.4)	48 (22.3)	52 (41.6)	
	Drinking, n (%)				0.015
	No	276 (81.2)	183 (85.1)	93 (74.4)	
	Yes	64 (18.8)	32 (14.9)	32 (25.6)	
Past medical history	Hypertension, n (%)				0.921
	No	254 (74.7)	161 (74.9)	93 (74.4)	
	Yes	86 (25.3)	54 (25.1)	32 (25.6)	
	Hyperlipidemia, n (%)				0.267
	No	277 (81.5)	179 (83.3)	98 (78.4)	
	Yes	63 (18.5)	36 (16.7)	27 (21.6)	
	Coronary heart disease, n (%)				0.720
	No	328 (96.5)	208 (96.7)	120 (96.0)	
	Yes	12 (3.5)	7 (3.3)	5 (4.0)	
	Fatty liver, n (%)				0.162
	No	282 (82.9)	183 (85.1)	99 (79.2)	
	Yes	58 (17.1)	32 (14.9)	26 (20.8)	
	Other diseases, n (%)				0.897
	No	327 (96.2)	207 (96.3)	120 (96.0)	
	Yes	13 (3.8)	8 (3.7)	5 (4.0)	
Sleep-related	Weekday sleep duration, n (%)				0.083
	≤6h	114 (33.8)	81 (38.0)	33 (26.6)	
	6-8h	187 (55.5)	109 (51.2)	78 (62.9)	
	≥8h	36 (10.7)	23 (10.8)	13 (10.5)	
	Weekend sleep duration, n (%)				0.483
	≤6h	54 (16.0)	38 (17.8)	16 (12.9)	
	6-8h	150 (44.5)	92 (43.2)	58 (46.8)	
	≥8h	133 (39.5)	83 (39.0)	50 (40.3)	
Physical activity	Physical activity level, n (%)				<0.001
	<600	72 (21.2)	69 (32.1)	3 (2.4)	
	600-1200	40 (11.8)	32 (14.9)	8 (6.4)	
	≥1200	228 (67.1)	114 (53.0)	114 (91.2)	
Physical indicators	BMI (kg/m²)	26.34 (22.13-30.55)	26.38 (22.21-30.55)	26.28 (21.98-30.58)	0.838
	Systolic Blood Pressure (mmHg)	130.0 (119.0-141.0)	130.0 (119.0-141.0)	129.0 (119.0-140.0)	0.478
	Diastolic Blood Pressure (mmHg)	75.0 (64.0-86.0)	75.0 (64.0-87.0)	74.0 (63.0-86.0)	0.491
	Pulse rate (bpm)	81.0 (74.0-90.0)	82.0 (74.0-90.0)	81.0 (73.0-90.0)	0.456
Glucose-related indicators	FPG (mmol/L)	9.19 (5.13-13.25)	9.18 (5.15-13.21)	9.20 (5.06-13.34)	0.971
	HbA1c (%)	8.51 (5.91-11.11)	8.39 (5.82-10.96)	8.72 (6.06-11.38)	0.272
Disease-related indicators	TC (mmol/L)	4.89 (3.67-6.11)	4.84 (3.68-6.00)	4.98 (3.65-6.31)	0.326
	TG (mmol/L)	2.62 (0.01-5.23)	2.52 (0.32-4.72)	2.80 (0.00-5.99)	0.376
	LDL-C (mmol/L)	2.97 (2.18-3.76)	2.97 (2.18-3.76)	2.97 (2.18-3.76)	0.984

Median (Q1, Q3); n (%); BMI, body mass index; TC, total cholesterol; TG, triglycerides; LDL-C, low-density lipoprotein cholesterol; FPG, fasting plasma glucose; HbA1c, hemoglobin A1c.

### 3.2 The association between sleep and glycemic control

**Figure 1:**
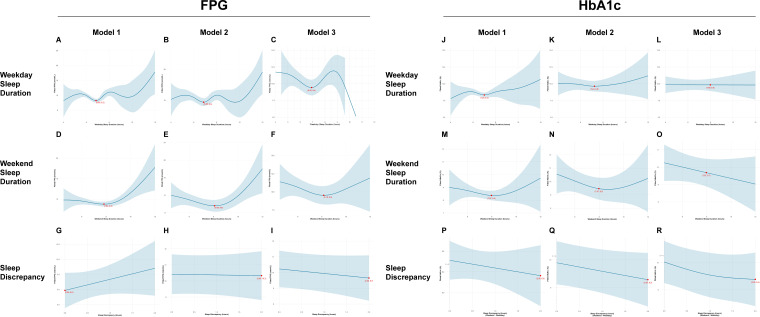
Association between sleep duration on weekdays/weekends and their difference with FPG and HbA1c. Smooth curves from generalized additive models depicting the associations of sleep duration with FPG and HbA1c.​ **(A-C)** represent the relationship between weekday sleep duration and FPG, **(D-F)** represent the relationship between weekend sleep duration and FPG, **(G-I)** represent the relationship between sleep discrepancy and FPG, **(J-L)** represent the relationship between weekday sleep duration and HbA1c, **(M-O)** represent the relationship between weekend sleep duration and HbA1c, and **(P-R)** represent the relationship between sleep discrepancy and HbA1c. The solid line represents the fitted curve, and the shaded area indicates the 95% confidence interval. The red dots mark the lowest points of the curve within the 6–8-hour range or at the minimum value of sleep difference. Model 1: Crude model (unadjusted). Model 2: Adjusted for gender, age, education level, profession, smoking, and alcohol consumption. Model 3: Further adjusted for body mass index (BMI), systolic blood pressure, diastolic blood pressure, total cholesterol (TC), triglycerides (TG), and low-density lipoprotein cholesterol (LDL-C);.

We conducted a GAM analysis, and the results showed that there was a significant non-linear association between weekday sleep duration and FPG (**Table 2**). In the unadjusted model (Model 1), this association was significant (edf = 5.1, p < 0.001). In the fully adjusted Model 3, this association still remained significant and explained about 30.0% of the variation in FPG (adjusted R² = 0.30). The curve shows a U-shaped association between the two. Within the 6–8-hour sleep range, the predicted FPG minimum corresponds to a sleep duration of 6.92 hours, at which time the predicted FPG is 9.4 mmol/L (95% CI: 8.2, 10.6). However, no significant non-linear association was found between weekday sleep duration and HbA1c (Model 3: edf = 1.0, p = 0.99). The results are presented in **Figure 1**. Furthermore, no significant nonlinear association was observed between weekend sleep duration and FPG or HbA1c (all p > 0.05) (**Table 2**). In the fully adjusted model, the association between weekend sleep duration and FPG was not significant (edf = 2.2, p = 0.19), and the association with between weekend sleep duration and HbA1c was also not significant (edf = 1.9, p = 0.26). The results are presented in **Figure 1**. The absolute difference of sleep duration between weekdays and weekends was not found to have a significant non-linear association with FPG and HbA1c (all p > 0.05) (**Table 1**). In the fully adjusted model, the associations between sleep difference and FPG (edf = 1.8, p = 0.33) and HbA1c (edf = 2.5, p = 0.13) did not reach statistical significance. The results are presented in **Figure 1**. Diagnostic checks for the GAM supported the adequacy of the specified smooth terms.

**Table 2:** GAM analysis of sleep duration and its difference on weekdays and weekends and glycemic control.

Exposure variable	Outcome variable	Model	edf	P-value	Adjusted R^2^	Minimum sleep duration/Time difference (hours)	Predicted value (95% CI)
Weekday sleep duration (h)	FPG (mmol/L)	**Model 1**	**5.1**	**<0.001**	**0.08**	**6.85**	**8.2 (7.6, 8.9)**
		**Model 2**	**5.1**	**<0.001**	**0.19**	**6.86**	**9.4 (8.1, 10.6)**
		**Model 3**	**3.5**	**0.02**	**0.30**	**6.92**	**9.4 (8.2, 10.6)**
	HbA1c (%)	Model 1	3.4	0.31	0.01	7.00	8.3 (8.0, 8.7)
		Model 2	2.1	0.58	0.17	7.24	9.6 (8.8, 10.4)
		Model 3	1.0	0.99	0.25	7.99	9.8 (8.9, 10.7)
Weekend sleep duration (h)	FPG (mmol/L)	**Model 1**	**2.9**	**0.01**	**0.03**	**7.82**	**8.8 (8.2, 9.4)**
		**Model 2**	**3.0**	**0.01**	**0.16**	**8.08**	**9.6 (8.3, 10.9)**
		Model 3	2.2	0.19	0.28	8.16	9.5 (8.3, 10.8)
	HbA1c (%)	Model 1	1.9	0.51	0.00	7.97	8.4 (8.0, 8.7)
		Model 2	1.9	0.26	0.17	7.97	9.5 (8.6, 10.3)
		Model 3	1.0	0.26	0.26	7.97	9.7 (8.9, 10.5)
Weekday minus weekend sleep duration (h)	FPG (mmol/L)	Model 1	1.0	0.22	0.00	0	9 (8.4, 9.6)
		Model 2	1.0	0.89	0.12	2	10.3 (8.8, 11.9)
		Model 3	1.0	0.33	0.27	2	9.7 (8.2, 11.1)
	HbA1c (%)	Model 1	1.0	0.44	0.00	2.00	8.3 (7.7, 8.9)
		Model 2	1.0	0.13	0.17	2.00	9.3 (8.3, 10.3)
		Model 3	1.0	0.13	0.26	2.00	9.4 (8.4, 10.4)

edf: effective degrees of freedom; 95% CI: confidence interval FPG: Fasting Plasma Glucose; HbA1c: Hemoglobin A1c.

Model 1: Crude model (unadjusted). Model 2: Adjusted for gender, age, education level, profession, smoking, and alcohol consumption. Model 3: Further adjusted for body mass index (BMI), systolic blood pressure, diastolic blood pressure, total cholesterol (TC), triglycerides (TG), and low-density lipoprotein cholesterol (LDL-C);.

edf is used to measure the degree of nonlinearity of the smoothing term, Bold values indicate statistical significance at p < 0.05.

### 3.3 The association between PA and glycemic control

We conducted a multiple linear regression to examine the association between PA and glycemic control. For the FPG, in the unadjusted model, the middle PA and high PA groups was on average about 2.81 and 3.54 units lower than that of the low PA group (**Table 3**). With further adjusting for BMI and five other metabolic indicators, the absolute values of the β coefficients slightly decreased (middle PA -2.76, high PA -3.22), but the association remained highly significant. This pattern could also be observed in the association between the HbA1c and PA.

**Table 3:** Multiple linear regression analysis of PA levels and glycemic control.

Metric	PA level	Model1		Model2		Model3	
		β(95%CI)	P-value	β(95%CI)	P-value	β(95%CI)	P-value
FPG							
	Middle PA	-2.81 (-4.31, -1.30)	**<0.001**	-3.24 (-4.72, -1.75)	**<0.001**	-2.76 (-4.14, -1.38)	**<0.001**
	High PA	-3.54 (-4.56, -2.52)	**<0.001**	-4.02 (-5.14, -2.90)	**<0.001**	-3.22 (-4.30, -2.14)	**<0.001**
HbA1c							
	Middle PA	-1.43 (-2.41, -0.44)	**0.005**	-1.84 (-2.78, -0.89)	**<0.001**	-1.50 (-2.49, -0.61)	**0.001**
	High PA	-1.80 (-2.48, -1.11)	**<0.001**	-2.17 (-2.90, -1.44)	**<0.001**	-2.01 (-2.76, -1.26)	**<0.001**

All statistical analyses used the Low PA group as the reference.

β, regression coefficient; CI, confidence interval; FPG, fasting plasma glucose; HbA1c, hemoglobin A1c; PA, physical activity

Model 1: Crude model (unadjusted). Model 2: Adjusted for gender, age, education level, profession, smoking, and alcohol consumption. Model 3: Further adjusted for body mass index (BMI), systolic blood pressure, diastolic blood pressure, total cholesterol (TC), triglycerides (TG), and low-density lipoprotein cholesterol (LDL-C).

Bold values indicate statistical significance at p < 0.05.

The median levels of FPG and HbA1c across PA groups are presented in **Table 4**. We conducted intergroup comparisons using significance levels adjusted with the Dunn-Bonferroni method. For FPG, the differences between the high PA and low PA groups, as well as between the medium PA and low PA groups, were highly significant (Adj. significance < 0.001), while the FPG levels of the high PA and medium PA groups were similar. This phenomenon can also be observed for HbA1c, except that the decreasing trend of medium PA compared to low PA had weaker statistical evidence than FPG (Adj. significance: 0.048). The results are presented in in **Table 4**. The results of multicollinearity diagnostics can be seen in **Supplementary Table 1**.

**Table 4:** Comparison of blood glucose levels across different PA level groups.

Metric	PA level	Median (Q1, Q3)	Overall P-value (H)	Pairwise comparisons (P-value)
FPG	Low PA	10.16 (8.34,15.64)	<0.001(H = 46.7)	Ref
	Middle PA	7.70 (6.60,10.50)		<0.001(vs. low)
	High PA	7.14 (6.30,9.13)		<0.001(vs. low),0.630(vs. moderate)
HbA1c	Low PA	9.80 (7.20,12.28)	<0.001(H = 22.5)	Ref
	Middle PA	7.70 (6.60,9.96)		0.048(vs. low)
	High PA	7.30 (6.30,9.30)		<0.001(vs. low),0.909(vs. moderate)

PA, Physical activity; FPG, fasting plasma glucose; HbA1c, hemoglobin A1c.

Data are expressed as median (first quartile, third quartile).; The Kruskal-Wallis H test was used for overall comparisons; Pairwise comparisons were conducted using Dunn’s *post hoc* test, with P-values adjusted by the Bonferroni correction.

### 3.4 Combined effects of sleep duration and PA on glycemic control

We conducted multiple linear regression analyses to examine the effects of different combinations of weekday and weekend sleep duration and physical activity levels on FPG and HbA1c. After gradually adjustment for demographic and clinical confounders (Model 3), compared with the “weekday short sleep & low PA” group, the “weekday moderate sleep & high PA” group was associated with the lowest FBG levels (β = -3.57, 95% CI: -4.99, -2.16, p < 0.001). In addition, “weekday moderate sleep & moderate PA” (β = -3.46, 95% CI: -5.45, -1.47, p < 0.001) and “weekday short sleep & high PA” (β = -2.65, 95% CI: -4.16 to -1.13, p < 0.001) also showed significantly lower FPG levels. The combined patterns of these groups also showed statistically significant associations with HbA1c. Other combinations of weekday sleep duration and physical activity levels compared with the reference group showed no statistically significant differences. The results are presented in in **Table 5**, and the complete results can be seen in **Supplementary Table 2**. The results of multicollinearity diagnostics can be seen in **Supplementary Table 1**.

**Table 5:** Associations of sleep-PA patterns with FPG and HbA1c.

Sleep-PA pattern	FPG (Model3)	HbA1c (Model3)
	β (95% CI)	β (95% CI)
Weekday		
Reference: Short sleep + Low PA		
Short sleep + Moderate PA	**−2.86(−4.96, −0.75)**	−1.34(−2.75, 0.08)
Short sleep + High PA	−2.65(−4.16, −1.13)*	**−1.45(−2.50, −0.41)**
Moderate sleep + Low PA	−1.31(−3.01, 0.38)	−0.18(−1.35, 1.00)
Moderate sleep + Moderate PA	−3.46(−5.45, −1.47)*	**−1.42(−2.75, −0.08)**
Moderate sleep + High PA	−3.57(−4.99, −2.16)*	−1.69(−2.67, −0.72)*
Long sleep + Low PA	−0.11(−2.45, 2.22)	−0.46(−2.12, 1.21)
Long sleep + Moderate PA	2.11(−1.35, 5.56)	2.19(−0.18, 4.55)
Long sleep + High PA	−1.98(−4.12, 0.16)	−1.29(−2.84, 0.26)
Weekend		
Reference: Short sleep + Low PA		
Short sleep + Moderate PA	−3.10(−6.32, 0.12)	−1.33(−3.42, 0.76)
Short sleep + High PA	**−3.04(−5.40, −0.68)**	**−1.80(−3.40, −0.20)**
Moderate sleep + Low PA	−1.58(−3.91, 0.76)	−0.72(−2.30, 0.86)
Moderate sleep + Moderate PA	−4.12(−6.67, −1.56)*	**−1.98(−3.68, −0.27)**
Moderate sleep + High PA	−3.54(−5.68, −1.39)*	**−1.89(−3.34, −0.44)**
Long sleep + Low PA	−1.26(−3.59, 1.06)	−0.81(−2.41, 0.78)
Long sleep + Moderate PA	−1.90(−4.73, 0.92)	−0.70(−2.61, 1.22)
Long sleep + High PA	−4.25(−6.39, −2.11)*	−2.25(−3.70, −0.80)*

PA, physical activity; FPG, fasting plasma glucose; HbA1c, glycated hemoglobin A1c; CI, confidence interval; β, regression coefficient.

Bold values indicate statistical significance at p < 0.05. * Indicate p < 0.001.

### 3.5 Sensitivity analysis

After excluding participants with extreme sleep durations (≤4 hours or ≥12 hours) (26), sensitivity analysis showed that the U-shaped association between weekday sleep duration and FPG remained significant (edf=0.393, P = 0.02), further support the stability of this association (**Supplementary Table 3**), as shown in **Supplementary Figure 1**. After regrouping according to the new PA criteria (low: <600 MET-min/week; moderate: 600-3000; high: ≥3000),we found that the results of the sensitivity analysis supported the findings of the main analysis. Both moderate and high-intensity activity were associated with significantly lower FPG compared with the low-intensity group (**Supplementary Table 4**).

## 4 Discussion

Our research systematically explored relationships among sleep duration, PA, and glycemic control in newly diagnosed T2DM patients. We discovered a significant U-shaped association between weekday sleep duration and FPG, suggesting the existence of a sleep range that maintains good glycemic control. Compared with individuals with low PA, those with moderate- and high-intensity PA showed significant improvements in blood glucose, but there was no difference between the two groups. There is a promising combined effect pattern between sleep and PA, and the combination of moderate-to-vigorous physical activity (MVPA) which might have good glycemic control effects. Moderate working day sleep & high PA and long weekend sleep & high PA respectively demonstrated good glycemic control.

### 4.1 Nonlinear association between sleep duration and glycemic control

Our research found that there exists a significant U-shaped relationship between sleep duration on working days and FPG through GAM. This discovery suggests​ that for newly diagnosed T2DM patients, both insufficient and excessive sleep may be associated with poorer glycemic control. In our analysis, the sleep duration associated with the lowest predicted FPG was approximately 6.9 hours on workdays. Recent studies have found that 7.32 hours of sleep on workdays is associated with the best glucose metabolism rate (27). A meta-analysis confirmed that compared with normal sleep (6–8 hours), both shorter (<6 hours) and longer (>8 hours) sleep durations are associated with poorer glycemic control (20), which is consistent with the results of another cohort study involving 2,962 diabetic patients that also supports this view (28).

However, while there was a significant U-shaped association between weekday sleep duration and FPG, the relationship between weekend sleep duration and FPG was not significant. This might be attributed to differences in daily routines (or sleep-wake patterns) between weekdays and weekends. The daily routine on weekdays is usually constrained by social requirements such as commuting times, making sleep patterns more regular and thus the impact of sleep duration on metabolism more consistent. However, sleep behavior on weekends could be more variable and often involves staying up late, sleeping in, sleep compensation or fragmented sleep, which disrupts the sleep rhythm. Regular circadian rhythms reduce β-cell compliance, thereby lowering glucose tolerance. But the disruption of sleep rhythms may decrease insulin sensitivity and exacerbate the body’s inflammation (29, 30). In addition to sleep duration, sleep quality can also be one of the factors influencing metabolism. Existing study suggested that the correlation between sleep quality and glycemic control in newly diagnosed T2DM (31). Sleep quality may play an important role in glycemic control when sleep duration can be freely determined on weekends. This study mainly measured sleep duration, while factors related to sleep quality were not included, which might be one reason for the insignificant difference in sleep between weekends and weekdays.

### 4.2 The association between PA and glycemic control

Daniel et al.’s meta-analysis concluded that for T2DM patients, the optimal intensity is 1,100 MET-hours per week and it could achieve a significant reduction in HbA1c (32). Our study further deepened the conclusion that PA might not only an independent protective factor for improving HbA1c but FPG in newly diagnosed T2DM. It is worth noting that there may exist a “plateau effect” in this protective factor. After adjusting for confounding factors, moderate-intensity PA didn’t show statistically significant additional benefits in reducing FPG and HbA1c compared to high-intensity PA.

This finding has significant clinical and public health implications. Although high-intensity PA has shown to have notable effect on controlling blood glucose and cardiovascular risks (33), but for newly diagnosed patients who have not developed a regular exercise habit yet, encouraging them to start with moderate-intensity physical activities such as walking and cycling might achieve ideal glycemic control (34). Compared to require them to do high-intensity exercise directly, a step-by-step approach can be more feasible and has a lower risk of injury. Lifestyle changes were crucial foundation for the management of prediabetes, and increasing physical activity frequency can achieve early glycemic control effects (35). PA not only has an evident effect on blood glucose management but also can prevent hypertension, dyslipidemia, cardiovascular diseases and other metabolic syndromes (36). The underlying mechanism might be that PA enhances the mitochondrial function in skeletal muscles, increasing mitochondrial capacity and energy metabolism (37), and alleviates insulin resistance in diabetic patients, thereby increasing insulin sensitivity (38). However, excessive activities can lead to mitochondrial dysfunction, reduce glucose tolerance and induce insulin secretion disorders (39). The reductions in HbA1c associated with moderate (-1.50%) and high (-2.01%) intensity PA in our cohort are clinically substantial. A large-sample meta-analysis revealed that in the case of the optimal physical activity dose of 1100 MET min/week, the reduction in HbA1c for patients with severely uncontrolled diabetes ranged from -1.02% to -0.66% (32). We posit several explanations. First, our cohort consisted of newly diagnosed T2DM patients. In this early stage of disease, patients may have better-preserved β-cell function and could be more responsive to lifestyle interventions like PA, leading to pronounced metabolic improvements (40). From a clinical perspective, a reduction in HbA1c is associated with better macrovascular and microvascular outcomes, including a lower risk of cardiovascular events, retinopathy, nephropathy, and other microvascular complications (41, 42). This indicates that adequate physical activity might be recognized as one of the core components of treatment strategies for newly diagnosed T2DM in future. Clinically, personalized exercise prescriptions can be formulated for these newly diagnosed patients to carry out targeted physical activity interventions. In the early stage of the disease, adopting regular and adequate physical activity is conducive to achieving ideal glycemic control, further emphasizing the essential clinical value of physical activity in the initial management of newly diagnosed T2DM.

### 4.3 The interaction effects of sleep and physical activity

This study also elucidated the combined associations of different sleep duration and PA levels on the glycemic control of newly diagnosed T2DM on working days and weekends. Data indicated that the combination of MVPA on both weekdays and weekends was associated with good glycemic levels. Compared with the unhealthy pattern of “short sleep duration and low-intensity PA”, merely prolonging sleep to normal duration but maintaining low activity did not demonstrate significant correlation in glycemic control. Specifically, “moderate weekday sleep duration and high-intensity PA” and “high weekend sleep duration and high-intensity PA” were associated with good glycemic control. These findings suggest that PA may amplify the benefits of sleep on glycemic control. As a result, whether it’s a weekday or a weekend, encouraging people to keep regular, sufficient sleep while maintaining at least moderate PA, preferably of high intensity, may maximize the improvement of glycemic control. This inclusion aligns with previous research indicating synergistic benefits of increased PA and sufficient sleep on glycemic control (43).

Interestingly, “short sleep and high-intensity PA” was also related with good glycemic control, offering a promising strategy for workers who are unable to sustain extended sleep duration to maintain glycemic control. When compared with weekdays, the weekend data showed that if the intensity of PA was sufficient (moderate or high), regardless of whether the sleep duration was at what level, most of the combinations are associated with better glycemic control.

The underlying potential mechanisms may be explained as follows. Insufficient sleep or disrupted circadian rhythms could disturb the hypothalamic-pituitary-adrenal axis, leading to imbalances in the body’s neuroendocrine, metabolic, and autonomic systems, and causing disorders in the secretion of hormones such as cortisol (44). While on the other hand, regular exercise could improve the body’s insulin sensitivity and promote the uptake of glucose by muscles (45). Meanwhile, PA can create a healthier environment for the metabolism of our body. Not only can it reduce the whole inflammation levels but also exert comprehensive protective effects on cardiovascular and various systemic organs (46), and adequate sleep can also enhance systemic immune defenses and reduce inflammation levels (47). Both jointly reduce the systemic inflammatory level, creating a metabolic anti-inflammatory environment for patients with T2DM. In fact, appropriate PA itself can significantly improve the quality of sleep at night, affecting sleep duration, efficiency and latency. Meanwhile, adequate rest boosts daytime energy and promotes a stronger willingness to be active, forming a virtuous cycle (48, 49). Future prospective or experimental studies are expected to more scientifically elucidate the relationship between PA and glycemic control, so as to provide mechanistic explanations and clinical guidance.

### 4.4 Contrasting patterns on weekdays and weekends

Our research revealed that there were differences in sleep duration and glycemic control outcomes between weekdays and weekends. There was a significant U-shaped association between sleep duration on weekdays and FPG, but it was not significant on weekends. However, MVPA was associated with good glycemic control both on weekdays and weekends.

The promising influence of social rhythms on sleep duration has been discussed in the previous text, that is, the regular constraints of the social rhythms on weekdays may make the relationship between sleep duration and FPG more stable and predictable. On the contrary, the complicated sleep patterns on weekends may break this correlation. Although the associated patterns of sleep vary, the connection between PA and glycemic control remained stable, and it was significant for both FPG and HbA1c. This might suggest that the association between PA and glycemic is less variable than sleep duration and could be a more important intervention element in the management of T2DM patients. However, the observational experimental design cannot fully confirm this. It is hoped that future prospective cohorts can further elaborate on this mechanism.

## 5 Strengths and limitations

### 5.1 Research strengths

This study has several advantages. We included newly diagnosed T2DM patients as our research subjects, for their potentially higher compliance with lifestyle interventions and were during a higher crucial intervention window, which can minimize confounding factors by excluding those on hypoglycemic drugs (50, 51). In the assessment of sleep duration, we refined the single measure of sleep duration into weekdays and weekends, and examined the relationship between sleep duration differences and blood glucose. Additionally, we systematically examined the independent and interactive effects of sleep and PA, gradually adjusted for confounding factors through multiple linear regression model and GAM, ensuring a rigorous approach. We combined the short-term blood glucose quantification index FPG with the long-term glycemic control index HbA1c for choosing outcome measures, making the research results more comprehensive. Focusing specifically on newly diagnosed patients represents a key strength of this study. This design minimized therapeutic confounding from long-standing disease or complex medication regimens, allowing us to isolate the intrinsic relationship between lifestyle factors and glycemic trajectories at disease onset. While these findings offer critical insights for early-stage intervention, we recognize the need to broaden their applicability. Moving forward, we aim to conduct multi-center studies to extend this research beyond the hospital setting and encompass the broader T2DM population.

### 5.2 Research limitations

This study also has several limitations. As a cross-sectional study, it can only suggest an association. Moreover, a key limitation lies in our reliance on self-reported sleep and physical activity data, rather than objective measures such as accelerometers. Although the International Sleep Diary and long-form IPAQ are widely validated, they remain prone to recall and social desirability biases (52, 53). Consequently, participants may have misreported sleep duration or overestimated activity levels, introducing misclassification. This measurement error likely resulted in residual confounding, which may have attenuated or exaggerated the true associations. And the relatively small sample size may compromise the statistical power of subgroup analyses and limit the generalizability of the findings to broader populations. Furthermore, there could be potential residual confounders not included in the current regressions. Dietary habits, for instance, exert a direct influence on glycemic control through total energy and carbohydrate intake. Similarly, our reliance on self-reported sleep duration overlooked sleep quality. The absence of data on psychological distress which may alter both self-care behaviors and physiological stress responses. Furthermore, while GAMs are useful for detecting nonlinear patterns, the estimated inflection points are model-dependent and should be interpreted with caution regarding their precise numerical value. This study employed a variety of statistical models to accommodate different data structures and research questions. Although this enhanced the depth of the analysis, it also made the presentation of results more complex. Future studies could collect these data to support robust estimates. At last, this study did not collect detailed metrics on sleep quality—such as insomnia, sleep apnea, or frequency of awakenings—which could have been more objectively assessed using the Pittsburgh Sleep Quality Index (PSQI) (54). Future research should adopt longitudinal designs, incorporate objective measurement instruments, collect and adjust several potential confounding factors that were not included and validate findings in larger, multi-center populations.

## 6 Conclusion

In conclusion, this study suggested that for newly diagnosed T2DM patients, maintaining a moderate sleep duration of approximately 7 hours on weekdays and engaging in at least moderate-intensity PA are lifestyle factors associated with​ better early glycemic control in newly diagnosed T2DM patients. It is particularly important that MVPA may play a crucial role in the combined effect of sleep and PA. On weekdays, the combination of “moderate sleep and high-intensity PA” should be emphasized; on weekends, the combination of “sufficient sleep and active engagement” yields the promising benefits. These findings provide a more scientific approach for the future management of T2DM.

## Data Availability

The data that support the findings of this study are available from the corresponding author, Dr. Siyu Dai, upon reasonable request.

## References

[1] American Diabetes Association. Classification and diagnosis of diabetes. Diabetes Care. (2015) 38:S8–S16. doi: 10.1002/9781119697473.ch2 25537714

[2] GBD 2021 Diabetes Collaborators . Global, regional, and national burden of diabetes from 1990 to 2021, with projections of prevalence to 2050: a systematic analysis for the Global Burden of Disease Study 2021. Lancet. (2023) 402:203–34. doi: 10.1016/s0140-6736(23)01301-6 37356446 PMC10364581

[3] Zhou YC Liu JM Zhao ZP Zhou MG Ng M . The national and provincial prevalence and non-fatal burdens of diabetes in China from 2005 to 2023 with projections of prevalence to 2050. Mil Med Res. (2025) 12:28. doi: 10.1186/s40779-025-00615-1 40457495 PMC12128495

[4] Duncan BB Magliano DJ Boyko EJ . IDF Diabetes Atlas 11th edition 2025: global prevalence and projections for 2050. Nephrol Dial Transplant. (2025) 41:7–9. doi: 10.1093/ndt/gfaf177 40874767

[5] Chan JCN Lim LL Wareham NJ Shaw JE Orchard TJ Zhang P . The Lancet Commission on diabetes: using data to transform diabetes care and patient lives. Lancet. (2021) 396:2019–82. doi: 10.1016/s0140-6736(20)32374-6 33189186

[6] Qiu ZX Qian F Zhang YB Liu J Geng TT Li R . Risk factor control in relation to mortality and life expectancy among people with type 2 diabetes: results from 3 nationwide cohort studies. Mil Med Res. (2025) 12:89. doi: 10.1186/s40779-025-00674-4 41351134 PMC12679727

[7] Chinese Diabetes Society . Guideline for the prevention and treatment of diabetes mellitus in China (2024 edition) (2025). Available online at: https://rs.yiigle.com/cmaid/1525739 (Accessed March 15, 2026).

[8] Schwarz P . IDF global clinical practice recommendations for managing type 2 diabetes - 2025. Diabetes Res Clin Pract. (2025) 222:112158. doi: 10.1016/j.diabres.2025.112158 40204550

[9] Rosenfeld RM Grega ML Karlsen MC Abu Dabrh AM Aurora RN Bonnet JP . Lifestyle interventions for treatment and remission of type 2 diabetes and prediabetes in adults: a clinical practice guideline from the American College of Lifestyle Medicine. Am J Lifestyle Med. (2025) 19:10s–131s. doi: 10.1177/15598276251325488 40546761 PMC12178993

[10] Rastogi S Pandey N Sachdev K . Linking Prameha etiology with diabetes mellitus: Inferences from a matched case-control study. Ayu. (2018) 39:139–45. doi: 10.1016/j.aimed.2019.03.363 31000990 PMC6454911

[11] Kramer CK Zinman B Retnakaran R . Short-term intensive insulin therapy in type 2 diabetes mellitus: a systematic review and meta-analysis. Lancet Diabetes Endocrinol. (2013) 1:28–34. doi: 10.1016/s2213-8587(13)70006-8 24622264

[12] Kim J Kim B Kim MK Baek KH Song KH Han K . Lifestyle changes and remission in patients with new-onset type 2 diabetes: a nationwide cohort study. J Korean Med Sci. (2025) 40:e24. doi: 10.3346/jkms.2025.40.e24 39995256 PMC11858603

[13] Taheri S Zaghloul H Chagoury O Elhadad S Ahmed SH El Khatib N . Effect of intensive lifestyle intervention on bodyweight and glycaemia in early type 2 diabetes (DIADEM-I): an open-label, parallel-group, randomised controlled trial. Lancet Diabetes Endocrinol. (2020) 8:477–89. doi: 10.1016/s2213-8587(20)30117-0 32445735

[14] Pikkemaat M Boström KB Strandberg EL . I have got diabetes!" - interviews of patients newly diagnosed with type 2 diabetes. BMC Endocr Disord. (2019) 19:53. doi: 10.1186/s12902-019-0380-5 31126267 PMC6534850

[15] Laiteerapong N Ham SA Gao Y Moffet HH Liu JY Huang ES . The legacy effect in type 2 diabetes: impact of early glycemic control on future complications (The Diabetes & Aging Study). Diabetes Care. (2019) 42:416–26. doi: 10.2337/dc17-1144 30104301 PMC6385699

[16] Adler AI Coleman RL Leal J Whiteley WN Clarke P Holman RR . Post-trial monitoring of a randomised controlled trial of intensive glycaemic control in type 2 diabetes extended from 10 years to 24 years (UKPDS 91). Lancet. (2024) 404:145–55. doi: 10.1016/s0140-6736(24)00537-3 38772405

[17] Kristensen FPB Sanchez-Lastra MA Dalene KE Del Pozo Cruz B Ried-Larsen M Thomsen RW . Leisure-time physical activity and risk of microvascular complications in individuals with type 2 diabetes: a UK Biobank study. Diabetes Care. (2023) 46:1816–24. doi: 10.2337/dc23-0937 37549380

[18] Rietz M Lehr A Mino E Lang A Szczerba E Schiemann T . Physical activity and risk of major diabetes-related complications in individuals with diabetes: a systematic review and meta-analysis of observational studies. Diabetes Care. (2022) 45:3101–11. doi: 10.2337/dc22-0886 36455117 PMC9862380

[19] Hou L Wang Q Pan B Li R Li Y He J . Exercise modalities for type 2 diabetes: a systematic review and network meta-analysis of randomized trials. Diabetes Metab Res Rev. (2023) 39:e3591. doi: 10.1002/dmrr.3591 36423199

[20] Lee SWH Ng KY Chin WK . The impact of sleep amount and sleep quality on glycemic control in type 2 diabetes: a systematic review and meta-analysis. Sleep Med Rev. (2017) 31:91–101. doi: 10.1016/j.smrv.2016.02.001 26944909

[21] Chinese Diabetes Society; National Office for Primary Diabetes Care . National guidelines for the prevention and control of diabetes in primary care (2022). Zhonghua Nei Ke Za Zhi. (2022) 61:249–62. doi: 10.3760/cma.j.cn112138-20220120-000063 35263966

[22] Carney CE Buysse DJ Ancoli-Israel S Edinger JD Krystal AD Lichstein KL . The consensus sleep diary: standardizing prospective sleep self-monitoring. Sleep. (2012) 35:287–302. doi: 10.5665/sleep.1642 22294820 PMC3250369

[23] Shan Z Ma H Xie M Yan P Guo Y Bao W . Sleep duration and risk of type 2 diabetes: a meta-analysis of prospective studies. Diabetes Care. (2015) 38:529–37. doi: 10.2337/dc14-2073 25715415

[24] Craig CL Marshall AL Sjöström M Bauman AE Booth ML Ainsworth BE . International physical activity questionnaire: 12-country reliability and validity. Med Sci Sports Exerc. (2003) 35:1381–95. doi: 10.1249/01.mss.0000078924.61453.fb 12900694

[25] Bull FC Al-Ansari SS Biddle S Borodulin K Buman MP Cardon G . World Health Organization 2020 guidelines on physical activity and sedentary behaviour. Br J Sports Med. (2020) 54:1451–2. doi: 10.1136/bjsports-2020-102955 33239350 PMC7719906

[26] Yin J Jin X Shan Z Li S Huang H Li P . Relationship of sleep duration with all-cause mortality and cardiovascular events: a systematic review and dose-response meta-analysis of prospective cohort studies. J Am Heart Assoc. (2017) 6(9):e005947. doi: 10.1161/jaha.117.005947 28889101 PMC5634263

[27] Fan Z Wei R Chen T Yan X Yin S Cao Y . Association of weekday sleep duration and estimated glucose disposal rate: the role of weekend catch-up sleep. BMJ Open Diabetes Res Care. (2026) 14:e005692. doi: 10.1136/bmjdrc-2025-005692 41775621 PMC12958941

[28] Kowall B Lehnich AT Strucksberg KH Führer D Erbel R Jankovic N . Associations among sleep disturbances, nocturnal sleep duration, daytime napping, and incident prediabetes and type 2 diabetes: the Heinz Nixdorf Recall Study. Sleep Med. (2016) 21:35–41. doi: 10.1016/j.sleep.2015.12.017 27448469

[29] Qian J Dalla Man C Morris CJ Cobelli C Scheer F . Differential effects of the circadian system and circadian misalignment on insulin sensitivity and insulin secretion in humans. Diabetes Obes Metab. (2018) 20:2481–5. doi: 10.1111/dom.13391 29862620 PMC6167165

[30] Leproult R Holmbäck U Van Cauter E . Circadian misalignment augments markers of insulin resistance and inflammation, independently of sleep loss. Diabetes. (2014) 63:1860–9. doi: 10.2337/db13-1546 24458353 PMC4030107

[31] Wen Z Ma M Zhang D Xiu L Jiang T . The relationship between PSQI scores and glucose metabolic dysfunction in patients with newly diagnosed T2DM: the mediating role of body composition. Diabetes Ther. (2025) 16:2111–22. doi: 10.1007/s13300-025-01789-6 40952648 PMC12579018

[32] Gallardo-Gómez D Salazar-Martínez E Alfonso-Rosa RM Ramos-Munell J Del Pozo-Cruz J Del Pozo Cruz B . Optimal dose and type of physical activity to improve glycemic control in people diagnosed with type 2 diabetes: a systematic review and meta-analysis. Diabetes Care. (2024) 47:295–303. doi: 10.2337/dc23-0800 38241499

[33] Li M Li J Xu Y Gao J Cao Q Ding Y . Effect of 5:2 regimens: energy-restricted diet or low-volume high-intensity interval training combined with resistance exercise on glycemic control and cardiometabolic health in adults with overweight/obesity and type 2 diabetes: a three-arm randomized controlled trial. Diabetes Care. (2024) 47:1074–83. doi: 10.2337/dc24-0241 38638032 PMC11116924

[34] Chen K Wang Y Li D Li J Huang Y Huang M . Impact of diverse aerobic exercise plans on glycemic control, lipid levels, and functional activity in stroke patients with type 2 diabetes mellitus. Front Endocrinol (Lausanne). (2024) 15:1389538. doi: 10.3389/fendo.2024.1389538 39359413 PMC11446103

[35] American Diabetes Association . Standards of medical care in diabetes--2010. Diabetes Care. (2010) 33:S11–61. doi: 10.2337/dc10-s011 20042772 PMC2797382

[36] Perry AS Dooley EE Master H Spartano NL Brittain EL Pettee Gabriel K . Physical activity over the lifecourse and cardiovascular disease. Circ Res. (2023) 132:1725–40. doi: 10.1161/circresaha.123.322121 37289900 PMC10254078

[37] Grevendonk L Connell NJ McCrum C Fealy CE Bilet L Bruls YMH . Impact of aging and exercise on skeletal muscle mitochondrial capacity, energy metabolism, and physical function. Nat Commun. (2021) 12:4773. doi: 10.1038/s41467-021-24956-2 34362885 PMC8346468

[38] Spartano NL Stevenson MD Xanthakis V Larson MG Andersson C Murabito JM . Associations of objective physical activity with insulin sensitivity and circulating adipokine profile: the Framingham Heart Study. Clin Obes. (2017) 7:59–69. doi: 10.1111/cob.12177 28112860 PMC5339058

[39] Flockhart M Nilsson LC Tais S Ekblom B Apró W Larsen FJ . Excessive exercise training causes mitochondrial functional impairment and decreases glucose tolerance in healthy volunteers. Cell Metab. (2021) 33:957–970.e6. doi: 10.1016/j.cmet.2021.02.017 33740420

[40] Zheng S Zhou H Han T Li Y Zhang Y Liu W . Clinical characteristics and beta cell function in Chinese patients with newly diagnosed type 2 diabetes mellitus with different levels of serum triglyceride. BMC Endocr Disord. (2015) 15:21. doi: 10.1186/s12902-015-0018-1 25924608 PMC4423127

[41] Kunutsor SK Balasubramanian VG Zaccardi F Gillies CL Aroda VR Seidu S . Glycaemic control and macrovascular and microvascular outcomes: a systematic review and meta-analysis of trials investigating intensive glucose-lowering strategies in people with type 2 diabetes. Diabetes Obes Metab. (2024) 26:2069–81. doi: 10.1111/dom.15511 38409644

[42] Dhami S Oli A . Study of correlation of severity of diabetic retinopathy with corneal thickness and endothelial parameter changes in diabetic patients. Rom J Ophthalmol. (2025) 69:567–74. doi: 10.22336/rjo.2025.87 PMC13065108 41971205

[43] Brakenridge CJ Koster A de Galan BE Carver A Dumuid D Dzakpasu FQS . Associations of 24 h time-use compositions of sitting, standing, physical activity and sleeping with optimal cardiometabolic risk and glycaemic control: the Maastricht Study. Diabetologia. (2024) 67:1356–67. doi: 10.1007/s00125-024-06145-0 38656371 PMC11153304

[44] Agorastos A Olff M . Sleep, circadian system and traumatic stress. Eur J Psychotraumatol. (2021) 12:1956746. doi: 10.1080/20008198.2021.1956746 34603634 PMC8480713

[45] Wanders L Gijbels A Bakker EA Trouwborst I Jardon KM Manusama KCM . Physical activity and sedentary behavior show distinct associations with tissue-specific insulin sensitivity in adults with overweight. Acta Physiol (Oxf). (2023) 237:e13945. doi: 10.1111/apha.13945 36745002

[46] Valenzuela PL Ruilope LM Santos-Lozano A Wilhelm M Kränkel N Fiuza-Luces C . Exercise benefits in cardiovascular diseases: from mechanisms to clinical implementation. Eur Heart J. (2023) 44:1874–89. doi: 10.1093/eurheartj/ehad170 37005351

[47] Irwin MR . Sleep and inflammation: partners in sickness and in health. Nat Rev Immunol. (2019) 19:702–15. doi: 10.1038/s41577-019-0190-z 31289370

[48] Sejbuk M Mirończuk-Chodakowska I Witkowska AM . Sleep quality: a narrative review on nutrition, stimulants, and physical activity as important factors. Nutrients. (2022) 14(9):1912. doi: 10.3390/nu14091912 35565879 PMC9103473

[49] Kredlow MA Capozzoli MC Hearon BA Calkins AW Otto MW . The effects of physical activity on sleep: a meta-analytic review. J Behav Med. (2015) 38:427–49. doi: 10.1007/s10865-015-9617-6 25596964

[50] Eisa N Barood O . Early combination therapy versus stepwise escalation in newly diagnosed type 2 diabetes: a systematic review and meta-analysis of multiple clinical outcomes. Can J Diabetes. (2026). doi: 10.1016/j.jcjd.2026.02.002 41707802

[51] Shen F Liu HY Liu YY Yang JQ Zhang LW Li QM . Accelerometer-derived physical activity associated with incidence and progression trajectory of cardiometabolic multimorbidity. Mayo Clin Proc. (2026). doi: 10.1016/j.mayocp.2025.11.020 41784584

[52] Zheng H Ang K Padmapriya N Kwan TK Moh MC Low S . Insights on accelerometer-measured 24-hour movement behaviour across type 2 diabetes sub-phenotypes in the Asian population. Diabetes Vasc Dis Res. (2026) 23:14791641261431769. doi: 10.1177/14791641261431769 41793152 PMC12967342

[53] Lauderdale DS Knutson KL Yan LL Liu K Rathouz PJ . Self-reported and measured sleep duration: how similar are they? Epidemiology. (2008) 19:838–45. doi: 10.1097/EDE.0b013e318187a7b0 PMC2785092 18854708

[54] de Menezes-Júnior LAA Carraro JCC MaChado-Coelho GLL Meireles AL . The Pittsburgh sleep quality index-2 (PSQI-2): the validity of a two-item sleep quality screener in Brazilian adults. Sleep Breath. (2025) 29:238. doi: 10.1007/s11325-025-03408-x 40637962

