# Supplementary material for: Sleep patterns, physical activity and glycemic control in newly diagnosed type 2 diabetes patients from a joint perspective: a cross-sectional study
Source: Front Endocrinol (Lausanne). 2026 Jun 17;17:1845188. doi: 10.3389/fendo.2026.1845188 (PMC13318745; doi:10.3389/fendo.2026.1845188)
Supplement: Supplementary file 1 [file DataSheet1.docx]

Supplementary Material

# Supplementary Figures and Tables

**Supplementary Table 1.** Variance Inflation Factor (VIF) of the multicollinearity assessment in the multiple linear regression model

|  | **PA main effect model** | | **weekday sleep duration-PA** | | **weekend sleep duration-PA** | |
| --- | --- | --- | --- | --- | --- | --- |
|  | **FPG** | **HbA1c** | **FPG** | **HbA1c** | **FPG** | **HbA1c** |
| **Independent variable / Interaction term** |  |  |  |  |  |  |
| Reference: Low PA | - | - | - | - | - | - |
| Moderate PA | 2.21 | 2.20 | - | - | - | - |
| High PA | 2.67 | 2.63 | - | - | - | - |
| Reference: Short sleep + Low PA |  |  |  |  |  |  |
| Short sleep + Moderate PA | - | - | 1.44 | 1.50 | 1.68 | 1.76 |
| Short sleep + High PA | - | - | 2.62 | 2.67 | 3.68 | 3.60 |
| Moderate sleep + Low PA | - | - | 1.79 | 1.85 | 3.38 | 3.38 |
| Moderate sleep + Moderate PA | - | - | 1.55 | 1.60 | 2.49 | 2.55 |
| Moderate sleep + High PA | - | - | 3.58 | 3.71 | 6.79 | 6.80 |
| Long sleep + Low PA | - | - | 1.29 | 1.28 | 3.57 | 3.34 |
| Long sleep + Moderate PA | - | - | 1.15 | 1.16 | 2.00 | 2.01 |
| Long sleep + High PA | - | - | 1.60 | 1.46 | 6.35 | 6.30 |
| **Sociodemographic variables** |  |  |  |  |  |  |
| Gender | 1.47 | 1.46 | 1.51 | 1.51 | 1.48 | 1.47 |
| Age | 2.20 | 2.20 | 2.22 | 2.23 | 2.24 | 2.26 |
| Education level | 1.94 | 1.94 | 1.93 | 1.95 | 1.98 | 2.00 |
| Profession | 1.54 | 1.52 | 1.53 | 1.50 | 1.50 | 1.49 |
| Smoking | 1.67 | 1.66 | 1.74 | 1.74 | 1.73 | 1.72 |
| Alcohol consumption | 1.53 | 1.52 | 1.54 | 1.55 | 1.54 | 1.55 |
| **Clinical metabolic variables** |  |  |  |  |  |  |
| BMI | 1.30 | 1.30 | 1.34 | 1.33 | 1.35 | 1.34 |
| SBP | 1.72 | 1.73 | 1.81 | 1.81 | 1.79 | 1.81 |
| DBP | 1.77 | 1.76 | 1.85 | 1.83 | 1.81 | 1.80 |
| TC | 8.68 | 8.74 | 9.01 | 9.03 | 8.98 | 8.98 |
| TG | 2.10 | 2.10 | 2.14 | 2.14 | 2.16 | 2.15 |
| LDL-C | 7.10 | 7.14 | 7.31 | 7.29 | 7.42 | 7.37 |

PA, Physical Activity; FPG, Fasting Plasma Glucose; HbA1c, Hemoglobin A1c; BMI, Body Mass Index; SBP, Systolic Blood Pressure; DBP, Diastolic Blood Pressure; TC, Total Cholesterol; TG, Triglycerides; LDL-C, Low-Density Lipoprotein Cholesterol.

**Supplementary Table 2.** The entire associations of sleep‑PA patterns with FPG and HbA1c

| **Sleep‑PA pattern** | **FPG** | | | **HbA1c** | | |
| --- | --- | --- | --- | --- | --- | --- |
|  | **β (95% CI) Model 1** | **β (95% CI) Model 2** | **β (95% CI) Model 3** | **β (95% CI) Model 1** | **β (95% CI) Model 2** | **β (95% CI) Model 3** |
| **Weekday** | | | | | | |
| Reference: Short sleep + Low PA |  |  |  |  |  |  |
| Short sleep + Moderate PA | **−3.21(−5.56, −0.85)**  **P=0.01** | **−2.82(−5.11, −0.53)**  **P=0.02** | **−2.86(−4.96, −0.75)**  **P=0.01** | **−1.61(−3.16, −0.06)**  **P=0.04** | **−1.45(−2.91, 0.01)**  **P=0.05** | −1.34(−2.75, 0.08)  P=0.06 |
| Short sleep + High PA | **−2.50(−4.06, −0.93)***  **P<0.001** | **−2.32(−3.91, −0.73)***  **P<0.001** | **−2.65(−4.16, −1.13)***  **P<0.001** | **−1.23(−2.30, −0.16)**  **P=0.02** | **−1.14(−2.19, −0.10)**  **P=0.03** | **−1.45(−2.50, −0.41)**  **P=0.01** |
| Moderate sleep + Low PA | −0.65(−2.54, 1.24)  P=0.51 | −0.51(−2.33, 1.32)  P=0.59 | −1.31(−3.01, 0.38)  P=0.13 | 0.10(−1.19, 1.381)  P=0.88 | 0.24(−0.96, 1.43)  P=0.67 | −0.18(−1.35, 1.00)  P=0.77 |
| Moderate sleep + Moderate PA | **−3.42(−5.54, −1.29)***  **P<0.001** | **−3.31(−5.37, −1.25)***  **P<0.001** | **−3.46(−5.45, −1.47)***  **P<0.001** | **−1.59(−3.01, −0.18)**  **P=0.03** | **−1.57(−2.90, −0.25)**  **P=0.02** | **−1.42(−2.75, −0.08)**  **P=0.04** |
| Moderate sleep + High PA | **−4.09(−5.52, −2.67)***  **P<0.001** | **−3.87(−5.34, −2.40)***  **P<0.001** | **−3.57(−4.99, −2.16)***  **P<0.001** | **−1.91(−2.88, −0.94)***  **P<0.001** | **−1.76(−2.73, −0.79)***  **P<0.001** | **−1.69(−2.67, −0.72)***  **P<0.001** |
| Long sleep + Low PA | 0.72(−1.77, 3.20)  P=0.57 | 0.84(−1.56, 3.24)  P=0.49 | −0.11(−2.45, 2.22)  P=0.93 | −0.39(−2.12, 1.34)  P=0.66 | −0.39(−2.00, 1.23)  P=0.639 | −0.46(−2.12, 1.21)  P=0.59 |
| Long sleep + Moderate PA | 1.34(−2.21, 4.89)  P=0.46 | 2.33(−1.10, 5.75)  P=0.18 | 2.11(−1.35, 5.56)  P=0.23 | 0.75(−1.64, 3.13)  P=0.53 | 1.23(−0.99, 3.45)  P=0.28 | 2.19(−0.18, 4.55)  P=0.07 |
| Long sleep + High PA | −1.88(−4.00, 0.25)  P=0.08 | **−2.16(−4.30, −0.03)**  **P=0.05** | −1.98(−4.12, 0.16)  P=0.07 | −1.27(−2.79, 0.25)  P=0.10 | −1.37(−2.82, 0.08)  P=0.06 | −1.29(−2.84, 0.26)  P=0.10 |
| **Weekend** | | | | | | |
| Reference: Short sleep + Low PA |  |  |  |  |  |  |
| Short sleep + Moderate PA | **−4.14(−7.78, −0.49)**  **P=0.03** | −3.09(−6.58, 0.40)  P=0.08 | −3.10(−6.32, 0.12)  P=0.06 | −1.94(−4.25, 0.37)  P=0.10 | −1.42(−3.57, 0.74)  P=0.20 | −1.33(−3.42, 0.76)  P=0.21 |
| Short sleep + High PA | **−3.56(−6.16, −0.96)**  **P=0.01** | **−3.24(−5.77, −0.70)**  **P=0.01** | **−3.04(−5.40, −0.68)**  **P=0.01** | **−2.06(−3.77, −0.34)**  **P=0.02** | **−1.74(−3.36, −0.11)**  **P=0.04** | **−1.80(−3.40, −0.20)**  **P=0.03** |
| Moderate sleep + Low PA | −1.58(−4.24, 1.07)  P=0.24 | −1.88(−4.41, 0.66)  P=0.15 | −1.58(−3.91, 0.76)  P=0.19 | −0.98(−2.74, 0.77)  P=0.27 | −0.97(−2.59, 0.66)  P=0.24 | −0.72(−2.30, 0.86)  P=0.37 |
| Moderate sleep + Moderate PA | **−4.42(−7.28, −1.57)***  **P<0.001** | **−4.69(−7.42, −1.95)***  **P<0.001** | **−4.12(−6.67, −1.56)***  **P<0.001** | **−2.67(−4.53, −0.80)**  **P=0.01** | **−2.59(−4.32, −0.86)***  **P=<0.001** | **−1.98(−3.68, −0.27)**  **P=0.02** |
| Moderate sleep + High PA | **−4.09(−6.49, −1.70)***  **P<0.001** | **−4.00(−6.32, −1.67)***  **P<0.001** | **−3.54(−5.68, −1.39)***  **P<0.001** | **−2.31(−3.89, −0.73)**  **P<0.001** | **−2.05(−3.54, −0.57)**  **P=0.01** | **−1.89(−3.34, −0.44)**  **P=0.01** |
| Long sleep + Low PA | −0.57(−3.19, 2.06)  P=0.67 | −0.49(−3.02, 2.04)  P=0.73 | −1.26(−3.59, 1.06)  P=0.29 | −0.88(−2.64, 0.87)  P=0.32 | −0.59(−2.22, 1.50)  P=0.48 | −0.81(−2.41, 0.78)  P=0.32 |
| Long sleep + Moderate PA | −1.60(−4.74, 1.55)  P=0.32 | −1.26(−4.25, 1.73)  P=0.41 | −1.90(−4.73, 0.92)  P=0.19 | −1.17(−3.25, 0.90)  P=0.27 | −0.75(−2.67, 1.16)  P=0.44 | −0.70(−2.61, 1.22)  P=0.47 |
| Long sleep + High PA | **−4.55 (−6.96, −2.13)***  **P<0.001** | **−4.62 (−6.95, −2.29)***  **P<0.001** | **−4.25(−6.39, −2.11)***  **P<0.001** | **−2.72(−4.31, −1.13)***  **P<0.001** | **−2.46(−3.95, −0.96)***  **P<0.001** | **−2.25(−3.70, −0.80)***  **P<0.001** |

PA, physical activity; FPG, fasting plasma glucose; HbA1c, glycated hemoglobin A1c; CI, confidence interval; β, regression coefficient.

Bold values indicate statistical significance at p < 0.05. * indicate p < 0.001.

**Supplementary Table 3.** Relationship between working day sleep duration and FPG after excluding extreme sleep

| **Exposure variable** | **Outcome variable** | **Model** | **edf** | **P-value** | **Adjusted R^2^** | **Minimum sleep duration / Time difference (hours)** | **Predicted value (95% CI)** |
| --- | --- | --- | --- | --- | --- | --- | --- |
| **Weekday sleep duration (h)** | **FPG (mmol/L)** | **Model1** | **3.7** | **<0.001** | **0.06** | **6.99** | **8.2(7.9, 9.0)** |
|  |  | **Model2** | **3.6** | **0.004** | **0.17** | **6.96** | **9.5(8.3, 10.8)** |
|  |  | **Model3** | **3.5** | **0.02** | **0.30** | **6.90** | **9.4(8.3, 10.6)** |

edf: effective degrees of freedom; 95% CI: confidence interval FPG: Fasting Plasma Glucose;

edf is used to measure the degree of nonlinearity of the smoothing term

Model 1: Crude model (unadjusted). Model 2: Adjusted for gender, age, education level, profession, smoking, and alcohol consumption. Model 3: Further adjusted for body mass index (BMI), systolic blood pressure, diastolic blood pressure, total cholesterol (TC), triglycerides (TG), and low-density lipoprotein cholesterol (LDL-C) ;

Bold values indicate statistical significance at p < 0.05.

**Supplementary Figure 1.** The entire associations of sleep‑PA patterns with FPG and HbA1c


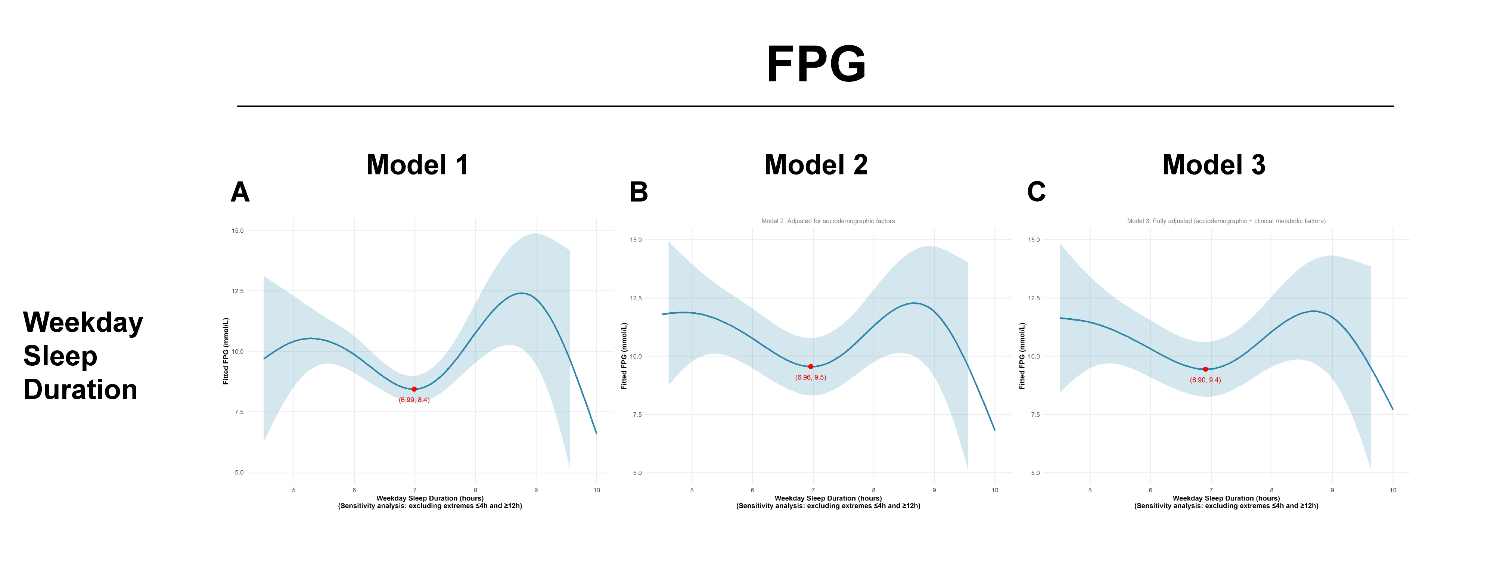


(A-C) represent the relationship between weekday sleep duration and FPG,

The solid line represents the fitted curve, and the shaded area indicates the 95% confidence interval. The red dots mark the lowest points of the curve within the 6-8-hour range.

**Supplementary Table 4.** Multiple linear regression analysis of adjusted physical activity groups and glycemic control

| **Metric** | **PA** | **Model1** | | **Model2** | | **Model3** | |
| --- | --- | --- | --- | --- | --- | --- | --- |
|  |  | **β(95%CI)** | **P-value** | **β(95%CI)** | **P-value** | **β(95%CI)** | **P-value** |
| **FPG** | | | | | | | |
|  | Middle PA | -3.79  (-4.84, -2.72) | **<0.001** | -3.46  (-4.62, -2.30) | **<0.001** | -2.88  (-4.08, -1.78) | **<0.001** |
|  | High PA | -2.83  (-4.00, -1.66) | **<0.001** | -2.99  (-4.39, -1.58) | **<0.001** | -2.71  (-4.08, -1.34) | **<0.001** |
| **HbA1c** | | | | | | | |
|  | Middle PA | -1.9  (-2.6, -1.2) | **<0.001** | -1.71  (-2.39, -0.97) | **<0.001** | -1.46  (-2.21, -0.72) | **<0.001** |
|  | High PA | -1.46  (-2.24, -0.67) | **<0.001** | -1.65  (-2.55, -0.97) | **<0.001** | -1.64  (-2.57, -0.71) | **0.001** |

All statistical analyses used the Low PA group as the reference

β, regression coefficient; CI, confidence interval; FPG, fasting plasma glucose; HbA1c, hemoglobin A1c; PA, physical activity

Model 1: Crude model (unadjusted). Model 2: Adjusted for gender, age, education level, profession, smoking, and alcohol consumption. Model 3: Further adjusted for body mass index (BMI), systolic blood pressure, diastolic blood pressure, total cholesterol (TC), triglycerides (TG), and low-density lipoprotein cholesterol (LDL-C).
